# Local adaptation and the potential effects of a contaminant on predator avoidance and antipredator responses under global warming: a space-for-time substitution approach

**DOI:** 10.1111/eva.12141

**Published:** 2014-01-06

**Authors:** Lizanne Janssens, Khuong Dinh Van, Sara Debecker, Lieven Bervoets, Robby Stoks

**Affiliations:** 1 Laboratory of Aquatic Ecology, Evolution and Conservation, University of LeuvenLeuven, Belgium; 2 Institute of Aquaculture, Nha Trang UniversityNha Trang, Vietnam; 3 Systemic, Physiological and Ecotoxicological Research Group, University of AntwerpAntwerp, Belgium

**Keywords:** antipredator traits, ecological risk assessment, escape speed, global warming, latitudinal gradient, metals, predator avoidance, space-for-time substitution, thermal adaptation

## Abstract

The ability to deal with temperature-induced changes in interactions with contaminants and predators under global warming is one of the outstanding, applied evolutionary questions. For this, it is crucial to understand how contaminants will affect activity levels, predator avoidance and antipredator responses under global warming and to what extent gradual thermal evolution may mitigate these effects. Using a space-for-time substitution approach, we assessed the potential for gradual thermal evolution shaping activity (mobility and foraging), predator avoidance and antipredator responses when *Ischnura elegans* damselfly larvae were exposed to zinc in a common-garden warming experiment at the mean summer water temperatures of shallow water bodies at southern and northern latitudes (24 and 20°C, respectively). Zinc reduced mobility and foraging, predator avoidance and escape swimming speed. Importantly, high-latitude populations showed stronger zinc-induced reductions in escape swimming speed at both temperatures, and in activity levels at the high temperature. The latter indicates that local thermal adaptation may strongly change the ecological impact of contaminants under global warming. Our study underscores the critical importance of considering local adaptation along natural gradients when integrating biotic interactions in ecological risk assessment, and the potential of gradual thermal evolution mitigating the effects of warming on the vulnerability to contaminants.

## Introduction

A key challenge for ecotoxicology is to understand how global warming will interact with contaminants to shape the *in situ* persistence of populations (Noyes et al. 2009; Hooper et al. 2013; Moe et al. 2013). Higher temperatures typically increase the toxicity of contaminants (Noyes et al. 2009; but see pyrethroids: Harwood et al. 2009) such as metals (Sokolova and Lannig 2008). Yet, a largely unresolved applied evolutionary question in this context is whether gradual thermal evolution will reduce or enlarge (through genetic trade-offs) the impacts of contaminants under global warming (Moe et al. 2013). To persist locally under global warming, animals will not only have to be able to deal with the temperature increase and with temperature-induced changes in interactions with anthropogenic stressors such as contaminants (Noyes et al. 2009), but also with the changed interactions with natural enemies such as predators (Gilman et al. 2010). At higher temperatures, prey may become more vulnerable to predators if the attack efficiency of the predator is increased relative to the escape efficiency of the prey (Kruse et al. 2008; De Block et al. 2013). An important consideration thereby is that contaminants may shape the prey's vulnerability to predation by affecting its activity levels, predator avoidance and antipredator responses (Mogren and Trumble 2010). Therefore, it is crucial to understand how contaminants will affect these behaviours under global warming and to what extent gradual thermal evolution may mitigate these effects.

Considerable progress has been made in the study of the effect of contaminants on activity levels, predator avoidance and antipredator responses (Mogren and Trumble 2010; Sornom et al. 2012; Cothran et al. 2013). Yet, how a temperature increase may modulate the effect of a contaminant on these fitness-related traits has rarely been studied (but see e.g. Broomhall 2004). The few experiments that focused on this used a ‘step-increase’ temperature experiment (De Frenne et al. 2013) at one latitude and therefore do not allow assessing the role of long-term gradual thermal evolution in mediating the impact of a temperature increase and the associated changes in sensitivity to contaminants. A key tool to evaluate the potential of long-term thermal evolution mitigating the effects of global warming at high latitudes is the space-for-time substitution approach (Dunne et al. 2004; Fukami and Wardle 2005; De Frenne et al. 2013). In this approach, animals from different latitudes are tested at two temperatures, whereby the projected temperature increase in the high-latitude sites matches the current temperature in the low-latitude sites (De Block et al. 2013). Such approach may inform us whether local thermal adaptation makes animals differentially vulnerable to contaminants, a key question for risk assessment at high latitudes under future climate scenarios (Moe et al. 2013).

In the current study, we assessed the vulnerability of an aquatic insect to a metal with regard to mobility, foraging, predator avoidance and antipredator responses under global warming. To test whether gradual thermal evolution may mitigate these effects at high latitudes, we applied a space-for-time substitution approach using a common-garden warming experiment with replicated populations from low and high latitudes spanning > 1500 km in Europe. This will allow evaluating whether local adaptation along natural gradients may interfere with the extrapolation of toxicity results from a single latitude across the species' range, which will be directly relevant for ecological risk assessment. Aquatic ecosystems are particularly vulnerable to global warming (Woodward et al. 2010) and to contamination (Bronmark and Hansson 2002) and are strongly shaped by predator–prey interactions (Wellborn et al. 1996). Warming effects on predator avoidance and antipredator traits of intermediate predators, such as damselfly larvae (Stoks and Cordoba-Aguilar 2012), in aquatic food webs may therefore increase our mechanistic understanding of how global warming may change aquatic food webs (Kratina et al. 2012). Damselflies are particularly relevant to study in this context as they are especially vulnerable to global warming (Hassall and Thompson 2008) and are showing among the strongest northward range shifts under global warming (Hickling et al. 2006). We focused on two general activities (mobility and foraging), predator avoidance (activity reductions under predation risk) and a key antipredator trait (escape swimming) of damselfly larvae (Stoks et al. 2003; Gyssels and Stoks 2005; Stoks and McPeek 2006). As contaminant, zinc was chosen as this metal is one of the most common contaminants in freshwater ecosystems (Brix et al. 2011), and it has been known to alter the predator avoidance and antipredator responses of aquatic species (Mogren and Trumble 2010). In a companion study, we documented that thermal adaptation shaped the zinc-induced mortality pattern with mortality only occurring when high-latitude larvae were exposed to zinc in the warming treatment (Dinh Van et al. 2013). We therefore expected similar signals of thermal adaptation on the chosen ecologically relevant and fitness-related behavioural traits.

## Materials and methods

### Study populations and rearing experiment

We studied populations of the damselfly *Ischnura elegans* (Vander Linden, Coenagrionidae) from the low-latitude (southern France) and high-latitude (southern Sweden) parts of the range in Europe (Dijkstra 2006) that were separated >1500 km. At each latitude, two randomly chosen populations were sampled, namely Salette (+45°43′30.58″N, +5°22′23.92″E) and Arandon (+45°42′35.64N, +5°25′47.28″E) for France; and Kalmar Dämme (56°40′9.84″N, 16°17′48.48″E) and Långviken (+56°39′11.88″N, +16°20′2.76″E) for Sweden. All populations were situated at shallow water bodies containing large dragonfly larvae (*Anax imperator*, Aeshnidae) as predators. Zinc concentrations in these populations were below detection limit (water: <2.5 *μ*g/L, sediment: <146 *μ*g/g dw sediment) as measured using inductively coupled plasma optical emission spectrometry (ICP-OES, Thermo scientific, ICAP 6300 Duo, Waltham, MA, USA).

In June 2010, we collected 10 mated females per population and placed them individually in small plastic vials with wet filter paper for oviposition. Eggs of each female were kept separately and were transferred to the laboratory in Belgium. Throughout the rearing experiment, eggs were incubated and larvae were reared at a water temperature of 20 or 24°C and a photoperiod of 16:8 h light/dark. Temperatures of 20 and 24°C reflect the mean summer water temperatures in shallow ponds in southern Sweden and southern France, respectively (De Block et al. 2013). Importantly, the 4°C temperature difference also corresponds with the predicted temperature increase by 2100 under IPCC scenario A1FI (IPCC 2007). Larvae were kept in group for the first 10 days to enhance survival (De Block and Stoks 2003). Thereafter, they were allocated individually to plastic vials (7.5 cm height, 3.5 cm diameter) filled to a height of 6 cm with dechlorinated tap water. Vials were placed in one of the three temperature-controlled water baths per rearing temperature and were regularly redistributed. All six water baths were placed in the same room to ensure equal condition such as light regimes. Larvae were fed *Artemia* nauplii *ad libitum* (459 ± 48, mean ± SE, *n* = 10) 5 days per week. When larvae moulted into the final instar, larvae from each rearing temperature were introduced in the exposure experiment at their respective temperature. Note that by doing so, all larvae had been fully acclimated to their experimental temperature (starting from the egg stage) before we tested the treatment effects**.**

### Experimental design

To test whether the effects of zinc exposure on activity, predator avoidance and antipredator responses depend on temperature and latitude of origin, we set up a full factorial experiment with 2 populations per latitude × 2 latitudes × 2 temperatures (20 and 24°C) × 3 zinc concentrations (0, 50, 150 mg zinc/L). Throughout the experiment, we did not keep track of female identity of the larvae but we randomly, and as equally as possible, distributed larvae of each female across the six combinations of temperature and zinc concentration. This avoided that potential differences in the responses among families (Hopkins et al. 2013) would interfere with the interpretation of the treatment effects. The zinc concentrations were based on a previous study on another coenagrionid damselfly (*Argia* sp.) that reported an LC_50 48 h_ of 320 mg/L (Wurtz and Bridges 1961). The chosen zinc concentrations are very high but have been observed in natural water bodies in contaminated areas in Europe (e.g. Nieto et al. 2007). Note that damselfly larvae are very resistant to zinc and that similar effects as the ones reported here are likely to occur at much lower zinc levels in other aquatic organisms that are more sensitive to zinc. We daily prepared the zinc solution based on a stock solution of ZnCl_2_ (5 g zinc/L dissolved in mQ water), which was stored in the dark at 4°C. We diluted the stock solution with synthetic pond water (for details see Janssens and Stoks 2013b), which was also used as control. Activity levels of damselfly larvae in this synthetic pond water do not differ from those observed in natural pond water (Janssens and Stoks 2013a).

At the start of the 6-day exposure period, final instar larvae were transferred individually to 100 mL glass jars. Jars were covered with brown tape on the sides to make sure that the larvae could not see each other (damselfly larvae are cannibalistic and impose predation threat upon each other, De Block and Stoks 2004). Each jar was filled with 50 mL of one of the three zinc solutions (0, 50 and 150 mg zinc/L). The medium in each jar was renewed daily to minimize the increase in zinc concentrations due to water evaporation from the jars. The measured zinc concentrations in the experimental vials with nominal concentrations of 50 and 150 mg zinc/L (based on a pooled sample of 10 vials) were 46 and 142 mg zinc/L when the medium was freshly renewed; after 24 h, zinc concentrations were 48 and 161 mg zinc/L at 20°C, and 49 and 154 mg zinc/L at 24°C, as verified with ICP-OES. During the exposure period, the larvae were fed daily the same amount of *Artemia* nauplii as during the pre-exposure period.

### Response variables

The activity of each larva was first scored in the absence and subsequently in the presence of chemical predator cues. After the activity test, the escape swimming speed was quantified. Each larva was tested at its rearing temperature. The activity test was run on day six of the exposure period and closely followed the protocol by Janssens and Stoks (2012). We observed the larval activity without predator cues first to avoid any lag effect of predator cues during the second observation period. Coenagrionid damselfly larvae do not show food saturation during the second observation period (Stoks and Johansson 2000; Stoks et al. 2005b; Janssens and Stoks 2012). Therefore, any difference in larval activity levels between the two observation periods can be attributed to predation risk. Prior to the activity test, each larva was weighted to the nearest 0.01 mg to correct for differences in individual body mass.

For the first observation period, each larva was acclimated for 7 min in a plastic container (15 cm × 10 cm × 12.5 cm) filled with 600 mL of medium corresponding to the zinc treatment of a given larva. As prey, *Artemia* nauplii (3560 ± 289, mean ± SE, *n* = 5) were then added to the container. We scored three larval activities for 7 min: general mobility (walking) and two traits directly linked to foraging (head orientations towards the prey and feeding strikes; Janssens and Stoks 2012). Walking was defined as the movement of a larva when at least one leg changed position; a head orientation was the movement of the head towards a prey item without moving the legs and a feeding strike was a quick extension of the labium towards a prey item. After 7 min, the larva was transferred to another container with the same medium, but with the addition of 1 mL predator medium. Again, a 7-min acclimation period was followed by a 7-min observation period.

We daily prepared the predator medium in a standardized way to keep the concentration of the chemical predator cues constant throughout the experiment. We had two plastic holding tanks (7.5 × 7.5 × 10 cm, filled with 400 mL of dechlorinated tap water), which each contained one larval *Anax imperator* dragonfly predator of the same size (ca. 30 mm). The water of these tanks was used to prepare the chemical cues. Two hours before the water extraction from these tanks, we fed each dragonfly one *I. elegans* damselfly larva which was always eaten within the first 10 min. After 2 h, we extracted ten ml water from each holding tank. In the pooled 20 mL volume, we homogenized one *I. elegans* larva and the resulting cocktail made up the predator medium that was added to the experimental damselfly larvae. Afterwards, we renewed the water in the holding tanks. Large aeshnid dragonfly larvae are important predators of *Ischnura* damselfly larvae (Stoks et al. 2005a), occur across the entire geographical distribution from France to Sweden (Dijkstra 2006) and were present in all four study populations. This way, damselfly larvae received a natural cocktail of predator kairomones and conspecific alarm cues to which they are known to react by reducing their activity levels (Stoks et al. 2003; Janssens and Stoks 2012).

Escape swimming speed was quantified directly after the activity test closely following the protocol of Stoks and McPeek (2006) using high-speed imaging. After the activity test, each larva was acclimated for 1 min in a white tray (20 cm × 15 cm × 4 cm) filled with 600 mL of dechlorinated fresh water at the rearing temperature of the larva. The larva was poked gently on the abdomen using a plastic pipette to mimic a predatory attack. Three successful swimming bouts were recorded using a high-speed camera (200 Hz, Basler AG, Ahrensburg, Hamburg, Germany) connected to a computer and controlled by Streampix 3 (NorPix Inc., Montreal, Quebec, Canada). Escape swimming speed (expressed in cm/s) was quantified based on the distance a larva swam during the first 0.2 s using the program Image-Pro Plus 5 (Media Cybernetics Inc., Warrendale, PA, USA). This initial response is most relevant for damselfly larvae to escape an attack from a sit-and-wait predator such as dragonfly larvae that do not pursue their prey after attacking it (Dayton et al. 2005). We used the average speed of three swimming bouts per larva for statistical analyses.

Sample sizes per response variable varied between 34 and 42 for each combination of latitude, zinc concentration and temperature, except for the treatment combinations with Swedish larvae tested at 24°C where sample sizes were lower (*n* = 13–14) due to higher mortality during the pre-exposure and exposure periods. Exact sample sizes per treatment combination are given in the Figures; the total number of larvae tested was 406 for activity levels and 379 for escape swimming speed.

### Statistical analyses

To test for effects of the zinc treatment, temperature and latitude on escape swimming speed, we ran an ancova using the mixed procedure of SAS v9.3 (SAS Institute Inc., Carry, NC, USA). Wet mass was included as a covariate. The analyses of the behavioural response variables were similar besides the fact that we considered the same behaviour measured in the absence and in the presence of predator chemical cues as repeats of that larva. Hence, we ran separate repeated-measures ancovas per behavioural response variable (walking, head orientations and feeding strikes). In these analyses, predation risk was the within-subject factor. All behaviours were log(x+1)-transformed to meet anova assumptions. Population nested in latitude was initially included as a random factor, but as it had no significant effect on any of the response variables, we removed it from the final models.

## Results

### Activity levels

**Figure 1:**
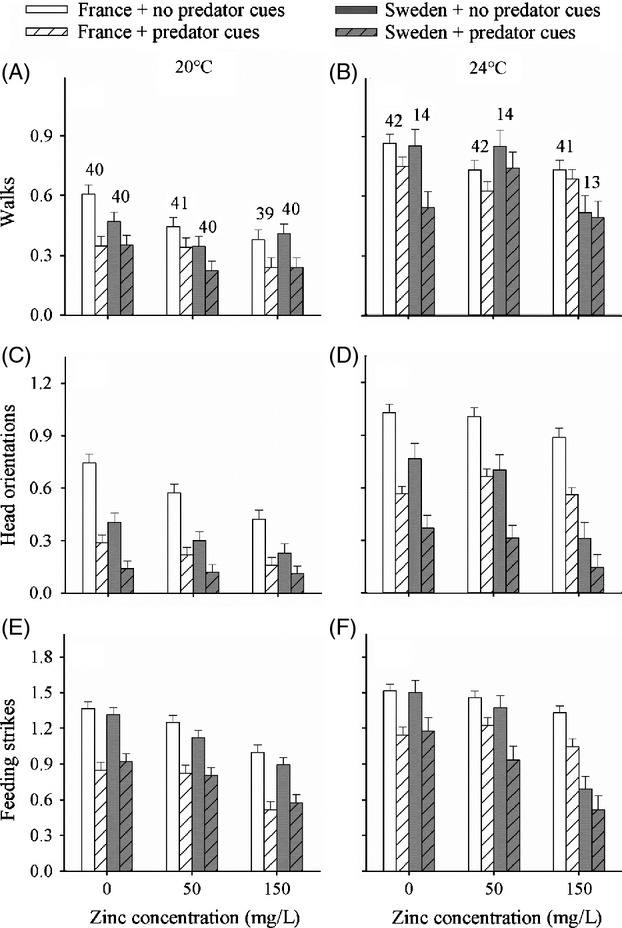
Mean (±SE) activity levels of *Ischnura elegans* larvae during the 6-day exposure period as a function of zinc concentration, temperature, latitude and predator cues: (A–B) number of walks, (C–D) head orientations and (E–F) feeding strikes. Given are least-square means of log(x + 1)-transformed data corrected for body mass. Sample sizes are above the bars. 24°C represents the mean summer water temperature in French populations, and 20°C represents the mean summer water temperature in the Swedish populations.

Overall, French larvae were more mobile and foraged more actively than Swedish larvae, and all three behaviours occurred more frequently at 24°C than at 20°C (main effects Latitude and Temperature, Table 1, Fig. 1). Both mobility and foraging activity were reduced with increasing zinc concentration (main effect Zinc, Table 1, Fig. 1). Moreover, at 24°C, these zinc-induced activity reductions were stronger in Swedish than in French larvae (Latitude × Temperature × Zinc, Table 1).

**Table 1:** The results of ancovas testing for the effects of latitude, temperature and zinc on activity levels and escape swimming speed of *Ischnura elegans* larvae. The successive behaviours scored in the absence and presence of predator cues were treated as repeats; ‘predator cue’ was the repeated factor.

	Walks			Head orientations			Feeding strikes			Escape swimming speed		
Effect	df1, df2	F	*P*	df1, df2	F	*P*	df1, df2	F	*P*	df1, df2	F	*P*
Latitude (Lat)	1, 393	3.15	0.073	1, 393	81.00	**<0.0001**	1, 393	17.32	**0.0002**	1, 379	47.36	**<0.001**
Temperature (Temp)	1, 393	88.66	**<0.0001**	1, 393	82.68	**<0.0001**	1, 393	37.04	**<0.0001**	1, 379	0.48	0.49
Zinc (Zn)	2, 393	7.93	**<0.0001**	2, 393	14.10	**<0.0001**	2, 393	33.31	**<0.0001**	2, 379	16.65	**<0.001**
Lat × Temp	1, 393	0.29	0.61	1, 393	5.56	**0.021**	1, 393	1.69	0.22	1, 379	2.32	0.13
Lat × Zn	2, 393	1.21	0.29	2, 393	0.41	0.61	2, 393	4.09	**0.016**	2, 379	3.34	**0.037**
Temp × Zn	2, 393	1.44	0.23	2, 393	1.30	0.23	2, 393	0.28	0.74	2, 379	1.11	0.33
Lat × Temp × Zn	2, 393	5.33	**0.006**	2, 393	3.82	**0.028**	2, 393	4.02	**0.021**	2, 379	0.15	0.86
Mass	1, 393	0.59	0.46	1, 393	0.73	0.37	1, 393	11.03	**0.001**	1, 379	16.80	**<0.001**
Predator cues	1, 393	8.47	**0.004**	1, 393	5.68	**0.019**	1, 393	11.69	**0.00065**			
Predator cues × Lat	1, 393	0.91	0.34	1, 393	11.18	**0.00085**	1, 393	1.19	0.28			
Predator cues × Temp	1, 393	3.48	0.063	1, 393	3.55	0.059	1, 393	8.68	**0.0034**			
Predator cues × Zn	2, 393	3.75	**0.024**	2, 393	9.30	**<0.0001**	2, 393	1.79	0.16			
Predator cues × Lat × Temp	1, 393	0.13	0.72	1, 393	2.29	0.13	1, 393	1.85	0.18			
Predator cues × Lat × Zn	2, 393	0.10	0.90	2, 393	0.84	0.52	2, 393	2.15	0.14			
Predator cues × Temp × Zn	2, 393	1.71	0.20	2, 393	0.23	0.87	2, 393	1.24	0.33			
Predator cues × Lat × Temp × Zn	2, 393	3.09	**0.045**	2, 393	0.92	0.32	2, 393	1.14	0.29			

Significant *P* values (*P* < 0.05) are indicated in bold.

Larvae reduced both their mobility and foraging activity in the presence of predator cues (main effect Predator cue, Table 1, Fig. 1). These predator-induced activity reductions depended upon latitude, temperature and zinc concentration. French larvae showed a more substantial predator-induced reduction in head orientations than Swedish larvae (Predator cue × Latitude, Table 1). All three behavioural antipredator responses depended upon temperature (Predator cue × Temperature, Table 1, Fig. 1) but not in a consistent way: the response in walking activity (trend) and feeding strikes was stronger at 20°C, while the response in head orientations tended to be stronger at 24°C. Overall, the predator-induced reductions in walking activity and head orientations were weaker in the presence of zinc (Predator cue × Zinc, Table 1, Fig. 1). For walking activity, this interactive effect was not present in Swedish larvae at 20°C generating a Predator cue × Latitude × Temperature × Zinc interaction (Table 1, Fig. 1A,B).

### Escape swimming speed

**Figure 2:**
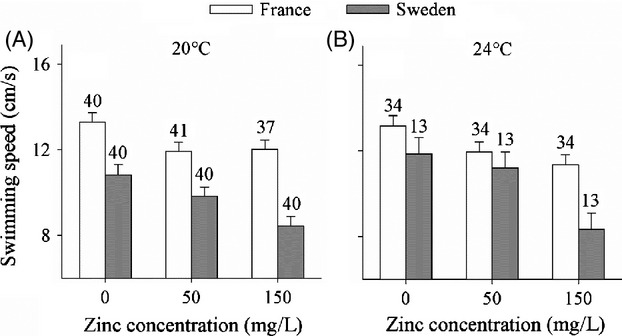
Mean (±SE) escape swimming speed of *Ischnura elegans* larvae as a function of zinc concentration, temperature and latitude. Given are least-square means corrected for body mass. Sample sizes are above the bars. 24°C represents the mean summer water temperature in French populations, and 20°C represents the mean summer water temperature in the Swedish populations.

French larvae swam faster than Swedish larvae (main effect Latitude, Table 1, Fig. 2). Exposure to zinc reduced swimming speed (main effect Zinc, Table 1, Fig. 2). This zinc-induced reduction was about twice as large in Swedish larvae (26%, control compared to 150 mg/L) than in French larvae (12%), as also indicated by a Latitude × Zinc interaction (Table 1).

## Discussion

### General activity levels

In line with the fact that multivoltine French larvae have less time per generation to complete larval development than semivoltine Swedish larvae, French larvae were more mobile and foraged more actively. In accordance, French larvae show a higher growth rate and have a shorter larval development compared with Swedish larvae (Stoks and De Block 2011; Stoks et al. 2012). Activity levels of larvae were higher at 24°C than at 20°C, reflecting the general pattern of higher activities at higher temperature in aquatic ectotherms (Stoks et al. 2014).

In agreement with studies on other taxa, damselfly larvae reduced all three scored behaviours when exposed to zinc, which can be explained by tissue damage, impairment of signal transduction, and/or the lowered energy content (Mogren and Trumble 2010). Reductions in the foraging-related traits likely negatively affect fitness as they will result in a lower food intake, hence may contribute to a zinc-induced reduction in larval growth rate (as was observed in the companion study, Dinh Van et al. 2013), an important fitness-related trait in damselflies (Stoks and Cordoba-Aguilar 2012). A slower life history in the presence of zinc may further enlarge the zinc effects in nature by extending the larval exposure period (e.g. Snodgrass et al. 2005).

A key finding was the stronger zinc-induced activity reduction for all three behaviours in Swedish larvae compared with French larvae when tested at 24°C. This pattern is consistent with differential thermal adaptation across latitudes. Indeed, the mean summer water temperature in shallow ponds in southern Sweden is 20°C, and therefore, larvae likely perceive 24°C, the mean summer water temperatures in shallow ponds in southern France, as a suboptimal temperature (De Block et al. 2013). We have shown before in this species that French larvae outperform Swedish larvae at 24°C in terms of growth rate and that this is genetically determined (Shama et al. 2011; Stoks et al. 2012). In line with this, we also observed a higher mortality during the pre-exposure period at 24°C in Swedish larvae in current study (see methods). This pattern of differential local thermal adaptation across latitudes likely caused the zinc to have a stronger effect on the activity levels at the higher temperature in Swedish larvae but not in French larvae. A similar pattern was observed for survival in the companion study: at the same concentrations, zinc did not cause mortality except in Swedish larvae at 24°C (Dinh Van et al. 2013). These observations illustrate that thermal adaptation may offset the pattern that metals have in general a stronger negative impact at higher temperatures (Sokolova and Lannig 2008) and that this is not only valid for lethal effects but also for sublethal effects on mobility and foraging activity.

### Predator avoidance and antipredator responses

Zinc exposure impaired key predator avoidance and antipredator responses in damselfly larvae: the predator-induced reductions in walking and head orientations were much less pronounced in the presence of zinc, and zinc caused a reduction in escape swimming speed. Reductions in activity levels and high escape speeds are widespread adaptive predator avoidance and antipredator responses to avoid being detected (e.g. Gyssels and Stoks 2005; Stoks et al. 2012; Cothran et al. 2013) and killed during an attack (Stoks and De Block 2000; Stoks and McPeek 2003), respectively. The impairment of adaptive predator avoidance and antipredator responses in animals exposed to metals is a widespread phenomenon (e.g. Lefcort et al. 2000; Brooks et al. 2009; McIntyre et al. 2012; Sornom et al. 2012) and has been explained by a reduced capacity to detect predators, an increased energy demand coupled with the inhibition of aerobic ATP production, and damage of neuromuscular transmission (e.g. Lurling and Scheffer 2007; Sokolova and Lannig 2008; Sornom et al. 2012). Such reduced responsiveness to chemical predator cues in the presence of metals may make prey more vulnerable to predation as shown for example in juvenile coho salmon exposed to copper (McIntyre et al. 2012). As the larvae exposed to zinc still had the lowest activity levels in the presence of predation risk, the reduced predator avoidance is unlikely to contribute to a higher vulnerability to predation. Yet, reduced escape speeds will result in a higher chance of being killed by attacking predators such as dragonfly larvae (Strobbe et al. 2009, 2010).

Another key finding was that the zinc-induced reduction in escape speed was more pronounced in Swedish larvae. This magnifies the latitudinal difference in escape speed with higher escape speeds in French larvae; the latter pattern matches the higher predator densities at low latitudes (R. Stoks P. Lambret and E. Svensson, unpublished data; see also Laurila et al. 2008). Possibly, the stronger predation pressures in French populations selected for better physiological defence mechanisms to avoid a too strong zinc-induced reduction in escape speed. Importantly, our findings indicate that zinc exposure would increase the vulnerability to attacking predators stronger in Swedish larvae compared with French larvae.

### Evolutionary perspectives with regard to global warming and ecological risk assessment

How organisms will cope with the changed interactions with contaminants as well as with predators will be a major factor in shaping the *in situ* persistence of their populations under global warming (Millennium Ecosystem Assessment 2005; Gilman et al. 2010; Sih et al. 2011; Urban et al. 2012). Moreover, as confirmed by our results, warming and contaminants may interact (Noyes et al. 2009) and contaminants such as metals may increase the vulnerability to predation. In a logical step forward, our study addressed the combined impact of warming and contaminants on activity, predator avoidance and antipredator responses at different latitudes. We will further highlight the here identified roles of local adaptation as a potential mediator of the effects of global warming and as an important factor to include in ecological risk assessment.

Our results tentatively suggest that in the absence of thermal evolution, hence under a scenario where Swedish larvae do not develop thermal adaptation to 24°C, and assuming all else staying equal, the predicted 4°C temperature increase by 2100 under IPCC scenario A1FI will make high-latitude populations more sensitive to contaminants such as zinc in terms of mobility and foraging activity compared with the current ambient summer water temperatures at the high latitude. Note that this, however, will critically depend on how warming will affect the susceptibility to zinc of the food and predators of the damselfly larvae. Moreover, it should be noted that increasing the temperature may also beneficially affect other traits of the Swedish populations. While it is highly unlikely (based on thermal degree days needed to complete a generation) that a 4°C warming will result in a switch from semivoltinism towards univoltinism at the high latitude, such temperature increase will reduce the exposure duration to the contaminant by shortening larval development times. A final aspect that needs consideration is the migration of low-latitude *I. elegans* genotypes to high-latitude populations. This is to be expected because odonates are among the taxa showing the strongest northward range shifts under global warming (Hickling et al. 2006), and strong gene flow occurs within the range of coenagrionid damselflies (Johansson et al. 2013). Our results suggest that invading low-latitude genotypes may perform better under global warming, especially in contaminated high-latitude sites, compared with the local high-latitude animals both during intraspecific competitive interactions as they show higher foraging levels and during interactions with invertebrate predators as they have a better escape swimming speed.

The used space-for-time substitution approach where the predicted 4°C temperature increase matches the current latitudinal difference in mean summer water temperatures of shallow water bodies inhabiting *I. elegans* between southern France and southern Sweden suggests that gradual thermal evolution may help the high-latitude populations to persist locally. This is based on the observation that the French larvae currently living at mean summer water temperatures of 24°C were less sensitive to zinc at 24°C than the Swedish larvae at 24°C, and on the assumption that the responses of the French currently living at 24°C are a good proxy for the expected responses of the Swedish larvae when they have enough time to adapt to a 4°C temperature increase by 2100.

Our findings highlight two implications for the role of local adaptation in ecological risk assessment. First, there is increasing awareness that impaired antipredator mechanisms may increase the negative effects of contaminants that are not captured in the typical single-species toxicity tests carried out in isolation (e.g. Brooks et al. 2009). The impairment of antipredator mechanisms has even been suggested as an index of heavy metal pollution (Lefcort et al. 2000). Our results add an important insight into these studies by demonstrating latitude-specific effects on the contaminant-induced impairment of antipredator mechanisms. This illuminates the critical importance of considering local adaptation along natural gradients when integrating biotic interactions in ecological risk assessment.

Second, whether local thermal adaptation makes animals differentially vulnerable to contamination is a key question for risk assessment of contaminants under future climatic scenarios (Moe et al. 2013). We identified large-scale latitudinal differentiation in the vulnerability of *I. elegans* damselfly larvae to zinc for the studied ecologically relevant traits with high-latitude populations being more sensitive in terms of swimming speed at both rearing temperatures and in terms of activity levels at the higher temperature. This pattern is opposite to the expectation that genetic adaptations to a warmer climate will come at the cost of a reduced tolerance to contaminants (Moe et al. 2013). This indicates that the assumed trade-off between tolerance to the warmer low-latitude climate and tolerance to contaminants may not be general. Importantly, the considerable latitude-dependent differences in vulnerability that we observed were repeatable across the two studied populations within a given latitude and occurred, while none of the populations had a history of metal contamination. While interpopulation differences in the vulnerability of animals to metals are well documented with regard to differences in local metal concentrations (reviewed in Morgan et al. 2007; Khan et al. 2011), little is known with regard to pristine habitats. In the only similar study, Cherkasov et al. (2010) documented the opposite pattern where oysters from two low-latitude populations were more sensitive to cadmium in terms of mitochondrial respiration than oysters from a high-latitude population. This study, together with ours, underscores the need to test contaminants at a large geographical scale and to evaluate their temperature-dependent impact across natural temperature gradients (Clements et al. 2012).

To conclude, our study illuminated the additional insights that can be obtained by assessing the impact of contaminants under global warming by using a space-for-time substitution approach. Combining a latitudinal gradient with a common-garden warming experiment not only revealed the potential of local adaptation to mitigate the effects of global warming in a contaminated world but also the need to consider local adaptation along natural gradients in ecological risk assessment. Space-for-time substitution studies have largely been neglected in ecotoxicology (Moe et al. 2013), and this despite the increasing awareness of the importance to consider evolution (Coutellec and Barata 2011; Jansen et al. 2011; Hammond et al. 2012; Cothran et al. 2013; Hopkins et al. 2013). More general, these substitution studies still are an underutilized tool in global change ecology (De Frenne et al. 2013). Our study illustrates the potential of this approach to explore topics at the interplay of both disciplines, and we advocate their addition to the standard toolbox of methods to study the role of evolution in ecotoxicology.

## Data archiving statement

Data for this study are available from the Dryad Digital Repository: http://dx.doi.org.10.5061/dryad.tn715.
